# Midkine promotes PDGF‐BB‐induced proliferation, migration, and glycolysis of airway smooth muscle cells via the PI3K Akt pathway

**DOI:** 10.14814/phy2.70553

**Published:** 2025-09-19

**Authors:** Yongtian Xu, Wenli Li, Jun Shi, Yanfang Guo

**Affiliations:** ^1^ Department of Pediatrics Gongli Hospital of Shanghai Pudong New Area Shanghai People's Republic of China; ^2^ Department of Pediatrics People's Hospital of Binchuan County Dali Yunnan People's Republic of China

**Keywords:** airway smooth muscle cells, glycolysis, Midkine, migration, PDGF‐BB, PI3K/Akt pathway, proliferation

## Abstract

Airway smooth muscle cell (ASMC) dysfunction drives airway remodeling in asthma, yet the molecular mediators remain incompletely defined. Midkine, a pleiotropic growth factor, has emerged as a potential regulator of inflammatory and proliferative responses. This study investigated midkine expression and function in platelet‐derived growth factor‐BB (PDGF‐BB)‐stimulated ASMCs. Serum midkine levels were quantified in asthmatic children and healthy controls. Primary human ASMCs were stimulated with PDGF‐BB and subjected to midkine knockdown using siRNA. Proliferation, migration, extracellular matrix (ECM) deposition, inflammatory cytokine production, glycolytic activity, and PI3K/Akt signaling were assessed using MTT, Transwell, RT‐qPCR, ELISA, western blotting, and metabolic assays. Serum midkine levels were significantly elevated in patients with asthma. PDGF‐BB stimulation increased midkine expression and ASMC proliferation, which were markedly suppressed by midkine silencing. Knockdown attenuated PDGF‐BB‐induced PI3K/Akt phosphorylation, reduced ASMC migration, and decreased collagen I and fibronectin expression levels. Midkine silencing also diminished TNF‐α, IL‐1β, and IL‐6 production and lowered glucose consumption, lactate release, ATP generation, and the expression of PKM2 and LDHA. These findings indicate that midkine promotes ASMC proliferation, migration, inflammation, extracellular matrix deposition, and glycolysis via the PI3K/Akt signaling pathway, thereby contributing to airway remodeling in asthma.

## 1 INTRODUCTION

Childhood asthma is one of the most prevalent chronic diseases affecting children globally, significantly contributing to mortality and morbidity rates (Akinbami & Schoendorf, 2002; Chung, 2016). This condition often results in frequent visits to emergency departments, hospital admissions, and reduced quality of life, thereby imposing a substantial burden on children, their families, and society at large (Cao et al., 2020). Current treatments involving inhaled corticosteroids can lead to various side effects, including hormone dependence or resistance, reduced bone mineral density, and stunted growth in children (Axelsson et al., 2019; Chen et al., 2018). Therefore, developing new therapeutic strategies to mitigate these adverse effects is essential.

Airway remodeling is a hallmark of chronic respiratory diseases, such as asthma, and is characterized by structural changes in the airway wall, including increased smooth muscle mass, epithelial alterations, and extracellular matrix (ECM) remodeling. The ECM not only provides structural support but also actively regulates cellular behavior via biochemical and mechanical signals. Excessive deposition and altered composition of ECM proteins, such as collagen, fibronectin, and elastin, contribute to airway wall thickening and stiffness, which exacerbate airway hyper‐responsiveness and airflow limitation (Holgate, 2008; Jeffery, 2001). Moreover, interactions between airway smooth muscle cells (ASMCs) and ECM facilitate pathological processes, including proliferation, migration, and inflammation, further driving airway remodeling (Kume, 2021; Wijsman et al., 2024). Therefore, understanding the molecular mediators that influence ECM dynamics is critical for developing targeted therapies for asthma and other chronic airway diseases.

Airway smooth muscle cells (ASMCs) are versatile cells that play a significant role in asthma pathogenesis (Prakash, 2013). The thickening of the airway smooth muscle during asthma progression is primarily attributed to ASMC hyperplasia. Recent findings suggest that the phenotypic flexibility of ASMCs, which shifts them toward a proliferative and synthetic state, is a key factor in cellular hyperplasia (Halwani et al., 2011). Platelet‐derived growth factor BB (PDGF‐BB) has been identified as a catalyst for various biological processes that promote ASMC proliferation and migration (Liu et al., 2015). Therefore, preventing ASMC transformation may hinder asthma development.

Midkine is a growth factor that binds to heparin and facilitates the movement of inflammatory cells, cytokine release, and inflammation (Krzystek‐Korpacka et al., 2011; Şalaru et al., 2016). It triggers Notch signaling (Kishida et al., 2013) and causes actin structural changes by interacting with the Notch‐2 receptor, leading to STAT3 phosphorylation and activation, which promotes cell growth and differentiation. By altering the Notch‐2 receptor, midkine activates various pathways and boosts the expression of Hes1 and NF‐κB (Kamakura et al., 2004). It is abundantly present in cystic pulmonary fibrosis and ARDS (Zhang et al., 2015). Recent research indicates that midkine influences airway inflammation and remodeling by affecting signaling pathways, such as the phosphoinositide 3‐kinase (PI3K)/Akt pathway, which is essential for cell survival, proliferation, and inflammatory responses (Şalaru et al., 2016; Zhao et al., 2019). The PI3K/Akt pathway is involved in the regulation of eosinophil activation, Th2 cytokine production, and smooth muscle hypertrophy, which are significant aspects of asthma (Cai et al., 2022). Furthermore, this pathway is vital for cellular metabolism, especially glycolysis, which supplies energy and biosynthetic precursors to rapidly dividing cells (Hoxhaj & Manning, 2020). Increased glycolysis in ASMCs is associated with hyperproliferative and migratory behaviors in asthma (Lv et al., 2025).

Given the role of midkine in promoting cell growth and the importance of the PI3K/Akt pathway in metabolic reprogramming, we hypothesized that midkine enhances PDGF‐BB‐induced proliferation, migration, and glycolysis in ASMCs via PI3K/Akt signaling. This study aimed to investigate whether midkine modulates PDGF‐BB‐driven ASMC dysfunction and to elucidate the involvement of the PI3K/Akt pathway in this process. Understanding this mechanism may provide new therapeutic targets for asthma by disrupting aberrant ASMC activation.

## 2 METHODS

### 2.1 Study population

This case–control study recruited 20 children with asthma between July 2022 and December 2024. We also collected 20 healthy children who underwent physical examinations (control group). Asthma was diagnosed based on the 2020 Asthma Guideline Update from the National Asthma Education and Prevention Program (Cloutier et al., 2020). The inclusion criteria were as follows: (1) meeting the diagnostic criteria for bronchial asthma; (2) first onset of illness; and (3) age <12 years. Exclusion criteria: (1) patients with any malignant hematological diseases or tumors; (2) patients with upper respiratory tract infections or those who received systemic corticosteroid treatment within 8 weeks. Based on the predefined inclusion and exclusion criteria, the study control group comprised 12 males and 8 females (mean age: 6.7 ± 2.4 years), while the asthma group included 11 males and 9 females (mean age: 7.1 ± 1.9 years). No statistically significant differences were observed between the groups in age, sex, or body mass index (BMI) (*p* > 0.05). However, a summary of the clinical characteristics of the study participants is provided in Table S1. This study was approved by the Ethics Committee of Shanghai Pudong New District Gongli Hospital (Approval No. GLYY1s2024‐052). All participants' parents or legally authorized guardians provided written informed consent to participate in the study. Fasting peripheral venous blood (5 mL) was collected from all participants to obtain serum.

### 2.2 Cell culture

Human ASMCs were purchased from ScienCell Research Labs (Cat#3400, Carlsbad, CA, USA) and cultured with high‐glucose DMEM (Cat#1965084, Gibco, Waltham, MA, USA) and 10% FBS (Cat#26140079, Gibco, Waltham, MA, USA), supplemented with 100 μg/mL streptomycin and 100 U/mL of penicillin (cat no. 15140122, Gibco, Waltham, MA, USA), ensuring the use of high‐quality reagents at 37 °C with 5% CO_2_. ASMCs were divided into four experimental groups: (1) Control group: Conventional culture without any treatment. (2) Model group (PDGF‐BB + si‐NC): Cells were transfected with negative control siRNA (si‐NC) for 48 h, followed by treatment with PDGF‐BB (Cat#PHG0041, Gibco, Waltham, MA, USA) (20 ng/mL) for 24 h to induce proliferation and phenotypic changes. (3) Midkine knockdown group (PDGF‐BB + si‐Midkine): Cells were transfected with si‐Midkine for 48 h to silence midkine expression, followed by stimulation with PDGF‐BB (20 ng/mL) for 24 h. This group was used to assess the role of midkine in PDGF‐BB–induced responses. (4) Midkine pathway inhibition with rescue treatment (PDGF‐BB + si‐Midkine + IGF‐1): Cells were transfected with si‐Midkine for 48 h to inhibit midkine signaling, followed by co‐treatment with IGF‐1 (Cat#ab270062, Abcam, UK) (100 ng/mL) and PDGF‐BB (20 ng/mL) for 24 h. This group was designed to evaluate whether pathway activation by IGF‐1 could reverse the effects of midkine knockdown.

### 2.3 Cell transfection

ASMCs in the logarithmic growth phase were seeded in 6‐well plates (1.0 × 10^5^ cells/well) until they reached 70%–80% confluence. Midkine siRNA (si‐Midkine) or its negative control (si‐NC) (GE Healthcare, Pittsburgh, PA, USA) was introduced into ASMCs using Lipofectamine 2000 (Cat#L3000001, Invitrogen, Carlsbad, CA, USA). Transfection efficiency was verified by RT‐qPCR and Western blotting 48 h after transfection.

### 2.4 MTT assay

ASMCs were seeded in 96‐well plates at a density of 1 × 10^4^ cells/well (Cat#2‐565‐135, Thermo Fisher Scientific, Waltham, USA). Subsequently, an MTT solution (1 mg/mL, Cat#M1020, Solarbio, and Beijing, China) was added to the cells and incubated for 4 h at 37°C. The resultant formazan crystals were dissolved in 100 μL of DMSO in each well. Finally, absorbance was measured at 570 nm using a microplate reader.

### 2.5 Enzyme‐linked immunosorbent assay (ELISA)

Serum samples were obtained from 20 individuals diagnosed with asthma and 20 healthy individuals. The concentrations of midkine (Cat#DY258, R&D Systems, Minneapolis, USA) in the serum were quantified. In addition, culture media from ASMCs were collected. The secretion levels of tumor necrosis factor‐alpha (TNF‐α) (Cat#DTA00D, R&D Systems, Minneapolis, USA), interleukin‐1 beta (IL‐1β) (Cat#DLB50, R&D Systems, Minneapolis, USA), and interleukin‐6 (IL‐6) (Cat#D6050B, R&D Systems, Minneapolis, USA) were assessed using enzyme‐linked immunosorbent assay (ELISA) kits (R&D Systems). Absorbance was measured at 450 nm using a microplate reader, and the concentrations were determined by referencing the appropriate standard curve.

### 2.6 Cell migration assay

A Transwell assay was used to assess the migration ability of ASMC. Transwell chambers (8 μm, 1 × 10^5^ cells/mL) were loaded with 100 μL of DMEM medium lacking FBS. The lower chamber was filled with 600 μL of DMEM containing 10% FBS. Following a 72‐h incubation, cells in the upper chamber were carefully removed with a cotton swab. To visualize the migrated cells, 0.1% crystal violet was applied. The average number of migrated cells was calculated by counting the stained cells in five different fields under a light microscope (Cat#415500‐0051‐000, CARL ZEISS, Germany) at a scale bar of 100 μm. Images were captured and processed using the ZEN 2.3 software (Carl Zeiss, Germany).

### 2.7 Detection of markers of glycolysis

Glucose consumption was evaluated using a Glucose Uptake Colorimetric Assay Kit (Cat#K676; BioVision, Mountain View, CA, USA). After transfection, the cells were incubated at 37°C for 48 h, harvested, and seeded into 96‐well plates at a density of 1 × 10^4^ cells per well. The cells underwent glucose deprivation through pre‐incubation for 40 min with 100 μL of Krebs‐Ringer‐Phosphate‐HEPES buffer, followed by a 20 min incubation with 10 μL of 2‐deoxyglucose (10 mM) and reaction mix A (for NADPH production) at 37°C for 1 h. Thereafter, the cells were treated with 90 μL of extraction buffer per well for 40 min at 90°C and subsequently placed in an ice bath for 5 min. Reaction Mix B (for recycling amplification) was then introduced to each well, and the mixture was centrifuged at 4°C and 16000×*g* for 2 min. The optical density of the supernatant was measured at 412 nm using a Varioskan LUX microplate reader (Thermo Fisher Scientific).

Lactate production was assessed in 6‐well plates, where 2 × 10^5^ cells were seeded and maintained at 37°C for 48 h using a Lactate Colorimetric Assay Kit (Cat#K627, BioVision). Subsequently, the cells were incubated in serum‐free medium at 37°C for an additional hour, after which the supernatant was collected for lactate production analysis. The reaction mixture was incubated at room temperature for 30 min in the dark, and the optical density of the supernatant was measured at 450 nm using a microplate reader (Varioskan LUX, Thermo Fisher Scientific).

ATP levels were quantified using an ATP Colorimetric Assay Kit (Cat#MAK1900; Sigma‐Aldrich; Merck KGaA) according to the manufacturer's instructions. Briefly, cells (5 × 10^5^) were combined with 100 μL of ATP Assay Buffer and centrifuged at room temperature for 5 min at 16000×*g*. The optical density of the supernatant was determined at 570 nm using a Varioskan LUX microplate reader (Thermo Fisher Scientific) (Suo et al., 2025).

### 2.8 Reverse transcription‐quantitative polymerase chain reaction (RT‐qPCR)

The PrimeScript RT Reagent Kit was purchased from TaKaRa Bio, Inc. (Cat#RR037A, TaKaRa Bio, Inc., Dalian, China) was used to synthesize complementary DNA (cDNA) (Cat#SK1241, Sangon Biotech, Shanghai, China) from 1 μg of total RNA extracted from ASMC cells. RNA extraction was conducted using TRIzol (Cat#15596018, Invitrogen, USA), a dependable product from Invitrogen. For reverse transcription quantitative polymerase chain reaction (RT‐qPCR), a Takara SYBR Green kit (Cat#1RR420, TaKaRa, Japan), known for its precision, was used in conjunction with an Applied Biosystems 7500 real‐time PCR machine (California, USA). The primer sequences used in this study are listed in Table 1, with GAPDH serving as the internal control. All primers were designed using the NCBI Primer‐BLAST Tool. The RT‐qPCR protocol commenced with an initial denaturation step at 95°C for 7 min, followed by 40 cycles of 15 s at 95°C and 1 min at 60°C. Relative mRNA levels were quantified using the 2^−ΔΔCt^ method (Zhu et al., 2023).

**TABLE 1:** List of primer sequences used in this investigation.

Genes	Forward primer (5′‐3′)	Reverse primer (5′‐3′)
Human Midkine	CGCGGTCGCCAAAAAGAAAG	TGCCCTCGCGGAAACCCACG
Human TNF‐α	CTGGGCAGGTCTACTTTGGG	CTGGAGGCCCCAGTTTGAAT
Human IL‐1β	CCAGGGACAGGATATGGAGCA	TTCAACACGCAGGACAGGTACAG
Human IL‐6	TCCACAAGCGCCTTCGGTC	GGTCAGGGGTGGTTATTGCAT
Human Collagen I	GAGGGCCAAGACGAAGACATC	CAGATCACGTCATCGCACAAC
Human Fibronectin	GATACCATCATCCCAGCTGTTC	CAGGAAGTTGGTTAAATCAATGGA
Human GAPDH	CATGTTGCAACCGGGAAGGA	CGCCCAATACGACCAAATCAG

### 2.9 Western blotting

Total proteins were isolated using RIPA lysis buffer (Cat#P0013B, Beyotime, Shanghai, China), and nuclear proteins were extracted using Extraction Reagents from Pierce Biotechnology, Inc. (Cat#PI78835, Rockford, IL, USA). Protein concentrations were determined using a BCA protein assay kit (Cat#P0012, Beyotime, Shanghai, China) from Beyotime Biotechnology. Protein samples (50 μg each) were separated by 10% SDS‐PAGE and subsequently transferred to PVDF membranes. These membranes were blocked with a 5% low‐fat milk solution and then incubated with primary antibodies against midkine (1:500, ab52637, rabbit monoclonal, Abcam), p‐PI3K (p85, Tyr458) (1:400, ab278545, rabbit monoclonal, Abcam), PI3K (1:500, ab302958, rabbit monoclonal, Abcam), p‐Akt (Ser473) (1:400, ab81283, rabbit monoclonal, Abcam), Akt (1:400, ab8805, rabbit polyclonal, Abcam), Collagen I (1:800, ab255809, rabbit monoclonal, Abcam), Fibronectin (1:600, sc‐8422, mouse monoclonal, Santa Cruz), TNF‐α (1:500, sc‐52746, mouse monoclonal, Santa Cruz), IL‐1β (1:400, sc‐12742, mouse monoclonal, Santa Cruz), IL‐6 (1:500, sc‐130326, mouse monoclonal, Santa Cruz), PKM2 (1:500, ab85555, rabbit polyclonal, Abcam), and LDHA (1:500, sc‐137243, mouse monoclonal, Santa Cruz). The membranes were then treated with a secondary antibody conjugated to horseradish peroxidase. GAPDH (1:2000, ab8245, mouse monoclonal, Abcam) was used as the internal control. Finally, the bands were visualized using ECL (Thermo Fisher Scientific, Cat#32109, Waltham, MA, USA) and analyzed using ImageJ software.

### 2.10 Statistical analysis

Data are presented as mean ± standard deviation (SD). Statistical analyses were performed using SPSS version 20.0 (SPSS, Chicago, IL, USA). To examine the differences among three or more groups, one‐way ANOVA was employed, followed by post hoc comparisons using the least significant difference (LSD) method. Statistical significance for one‐tailed tests was defined as *p* < 0.05, *p* < 0.01, and *p* < 0.001.

## 3 RESULTS

### 3.1 Midkine is elevated in asthma and promotes PDGF‐BB‐induced ASMC proliferation

**FIGURE 1:**
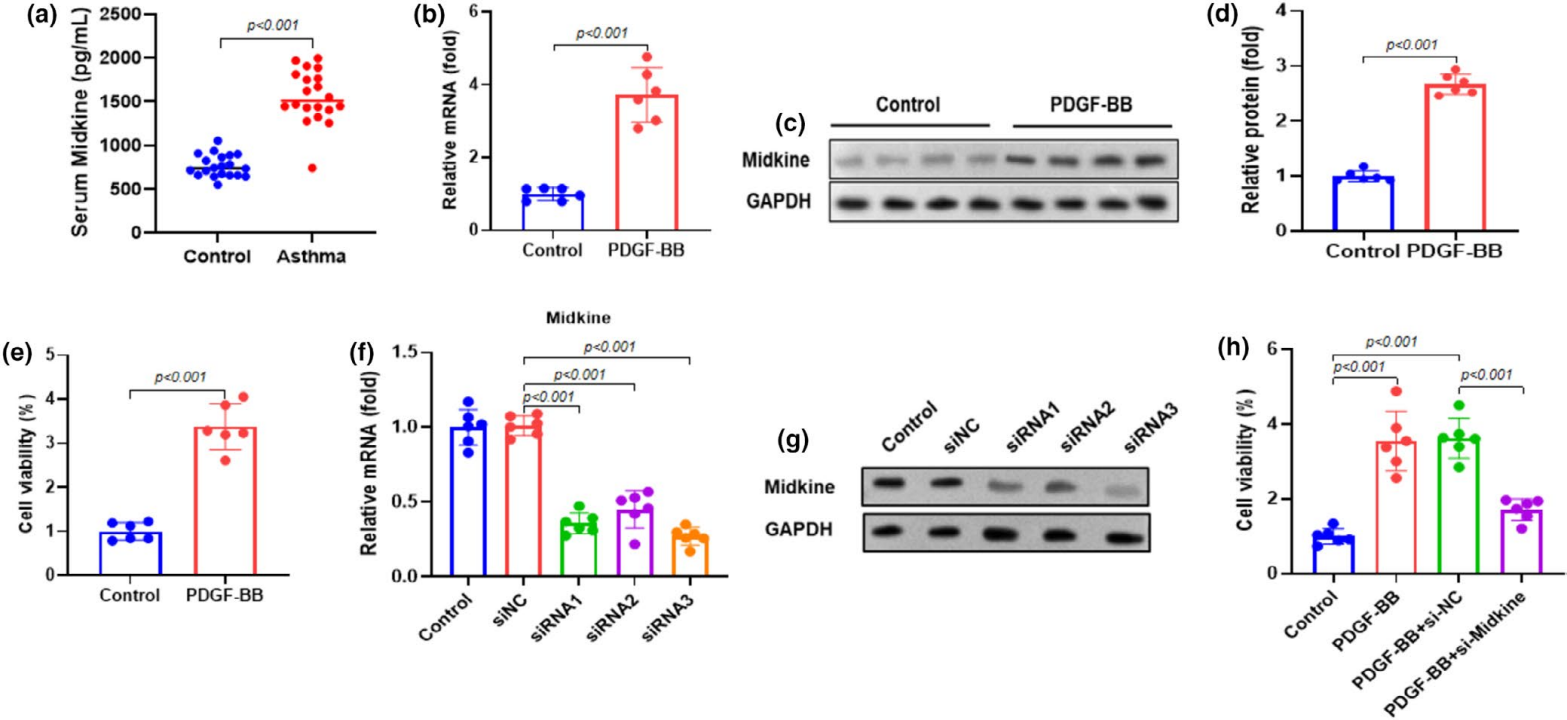
Midkine was up‐regulated in asthma. (a) Serum midkine levels were higher in asthmatic children than in the control group (*n* = 20 in each group). Human ASMCs were treated with 0 or 20 ng/mL PDGF‐BB for 24 h. (b) Midkine mRNA expression was determined using RT‐qPCR. (c) Midkine protein expression was determined by western blotting. Representative bands are shown in the figure. (d) Quantification of midkine protein in ASMCs relative to GAPDH. (e) Cell viability of ASMCs was determined using the MTT assay. Midkine was knocked down in ASMCs, and midkine siRNA (si‐midkine) and control siRNA (si‐NC) were transfected to ASMCs for 48 h. (f) RT‐qPCR was performed to assess midkine mRNA expression. (g) Western blotting was performed to assess the expression of midkine protein. Sequence #3 showed the best inhibitory effect and was chosen for subsequent experiments. (h) ASMCs were transfected with si‐midkine or si‐NC, followed by incubation with 20 ng/mL PDGF‐BB for 24 h. Data are presented as mean ± SD from at least three independent biological replicates, each performed in 6 duplicate wells, and analyzed using an unpaired t‐test.

Serum midkine levels were markedly elevated in asthmatic children compared to healthy controls (*n* = 20 per group) (Figure 1a). In human ASMCs, treatment with PDGF‐BB (20 ng/mL, 24 h) significantly increased midkine mRNA and protein expression compared to untreated cells (Figure 1b–d) and enhanced cell viability, as determined by the MTT assay (Figure 1e). To explore the functional role of midkine, ASMCs were transfected with midkine siRNA (si‐midkine) or control siRNA (si‐NC) for 48 h. RT‐qPCR confirmed efficient midkine knockdown, with the #3 siRNA sequence producing the most pronounced suppression and therefore selected for subsequent experiments (Figure 1f). Western blot analysis further verified the decrease in midkine protein expression following siRNA transfection (Figure 1g). Upon stimulation with PDGF‐BB (20 ng/mL, 24 h), midkine knockdown significantly reduced ASMC proliferation compared with that in si‐NC, as shown by the MTT assay (Figure 1h). Collectively, these findings indicate that midkine is upregulated in asthma and promotes ASMC proliferation, an effect that can be suppressed by midkine knockdown.

### 3.2 Midkine knockdown suppressed PDGF‐BB‐induced PI3K/Akt activation in ASMCs

**FIGURE 2:**
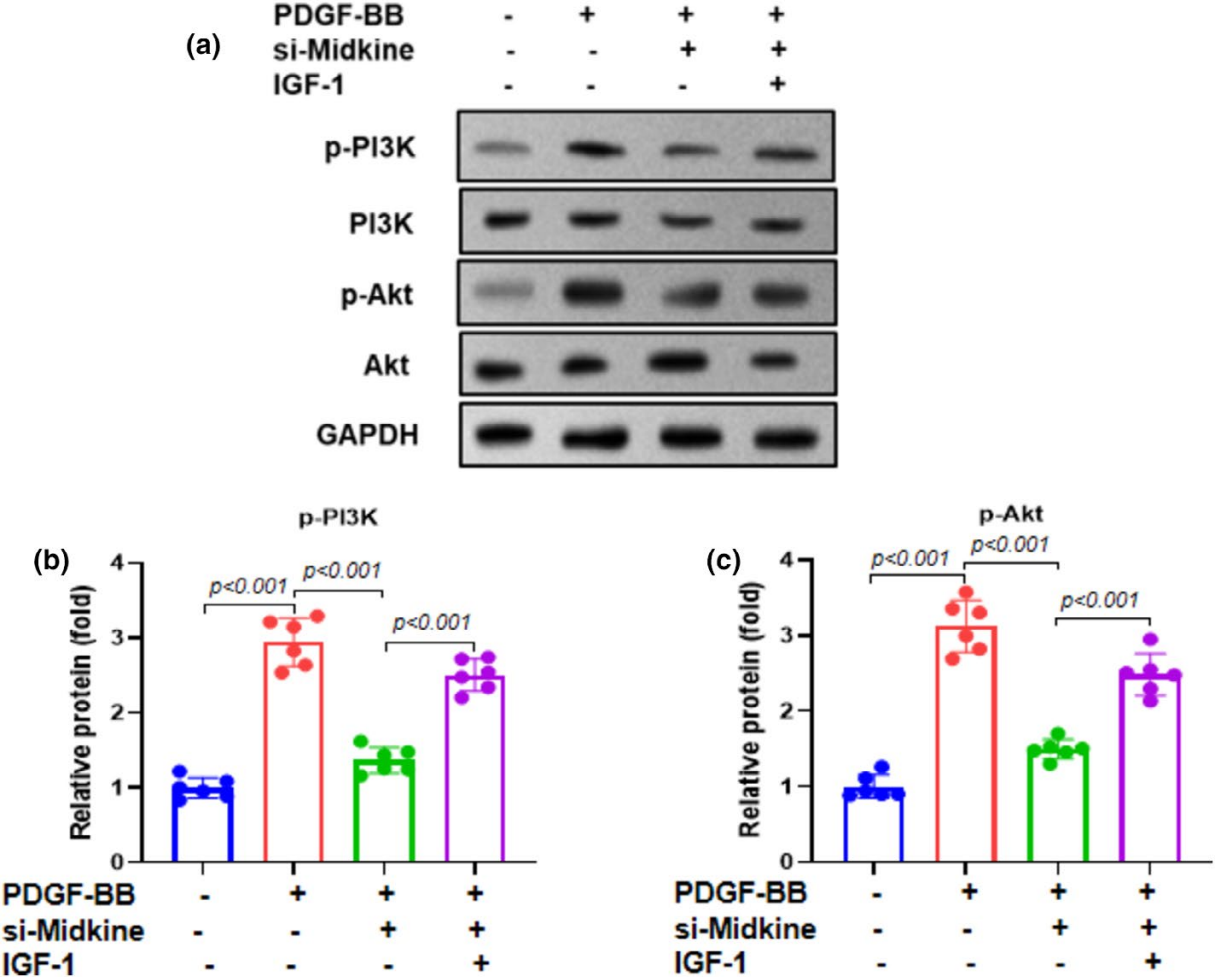
Midkine knockdown inhibits the PI3K/Akt pathway in PDGF‐BB‐treated ASMCs. ASMCs were transfected with si‐midkine for 48 h and then incubated with the PI3K/Akt pathway activator IGF‐1 (100 ng/mL) and PDGF‐BB (20 ng/mL) for a further 24 h. (a) Representative gel blots depicting the levels of phosphorylated PI3K and phosphorylated Akt. (b, c) Quantification of p‐PI3K and p‐Akt protein band intensities. Data are presented as mean ± SD from at least three independent biological replicates, each performed in six duplicate wells, and analyzed using one‐way ANOVA, followed by Bonferroni post‐hoc test.

To assess the involvement of midkine in PDGF‐BB‐induced PI3K/Akt signaling, ASMCs were transfected with si‐midkine for 48 h and subsequently treated with PDGF‐BB (20 ng/mL) in the presence or absence of the PI3K/Akt pathway activator IGF‐1 (100 ng/mL) for 24 h. Western blot analysis demonstrated that silencing midkine markedly reduced the phosphorylation of PI3K (p‐PI3K, p85, Tyr458) and Akt (p‐Akt, Ser473) compared to the control groups (Figure 2a). Densitometric analysis confirmed that PDGF‐BB‐induced phosphorylation of PI3K (Figure 2b) and Akt (Figure 2c) was significantly attenuated in the si‐midkine‐transfected cells. These results indicate that midkine is essential for PDGF‐BB‐mediated activation of the PI3K/Akt signaling pathway.

### 3.3 Midkine knockdown inhibits PDGF‐BB‐induced ASMC migration and ECM protein expression

**FIGURE 3:**
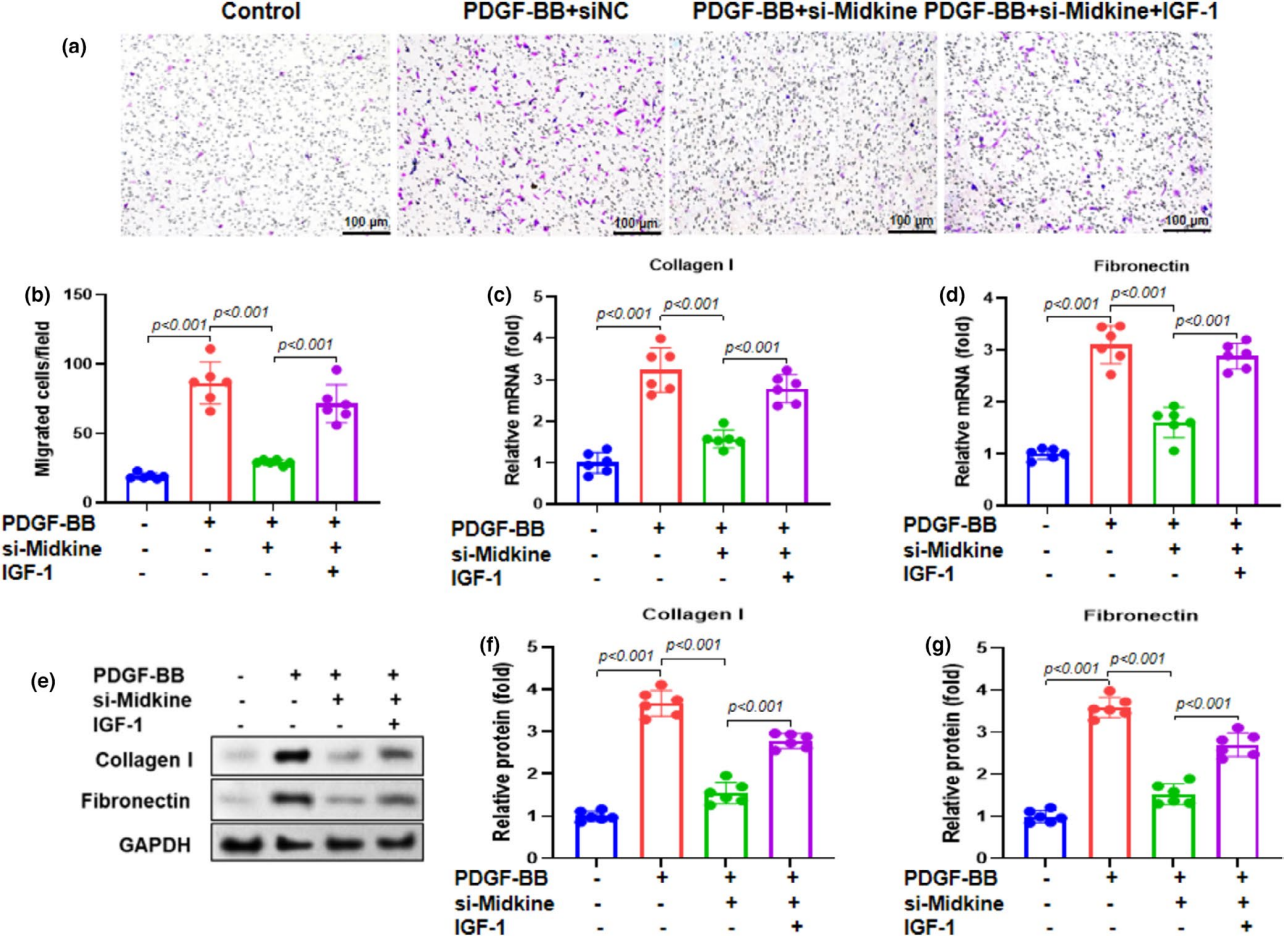
Midkine knockdown inhibits migration of PDGF‐BB‐treated ASMCs. (a) Transwell assay was used to assess the migration ability of ASMCs with 0.1% crystal violet staining (scale bar, 100 μm). (b) Quantification of the number of migrating cells. RT‐qPCR was used to assess the mRNA expression of (c) Collagen I and (d) fibronectin. (e) Western blotting was used to assess the protein expression of Collagen I and fibronectin. Representative bands are shown. (F, G) Quantification of Collagen I and fibronectin in ASMCs. Data are presented as mean ± SD from at least three independent biological replicates, each performed in six duplicate wells, and analyzed using one‐way ANOVA followed by Bonferroni post‐hoc test.

Midkine knockdown markedly inhibited PDGF‐BB‐induced ASMC migration. Transwell migration assays revealed a pronounced reduction in the number of migrated cells following midkine silencing, as visualized by 0.1% crystal violet staining (Figure 3a) and confirmed by quantitative analysis (Figure 3b). RT‐qPCR demonstrated that midkine knockdown significantly downregulated the expression of extracellular matrix (ECM) components, including collagen I (Figure 3c) and fibronectin (Figure 3d). Western blot analysis showed a consistent pattern, with reduced collagen I and fibronectin protein levels (Figure 3f,g). These results indicate that midkine contributes to ECM remodeling in ASMCs, and its silencing suppresses PDGF‐BB‐induced migration and ECM protein expression, highlighting its potential role in airway remodeling.

### 3.4 Midkine knockdown suppressed PDGF‐BB‐induced proinflammatory cytokine production in ASMCs

**FIGURE 4:**
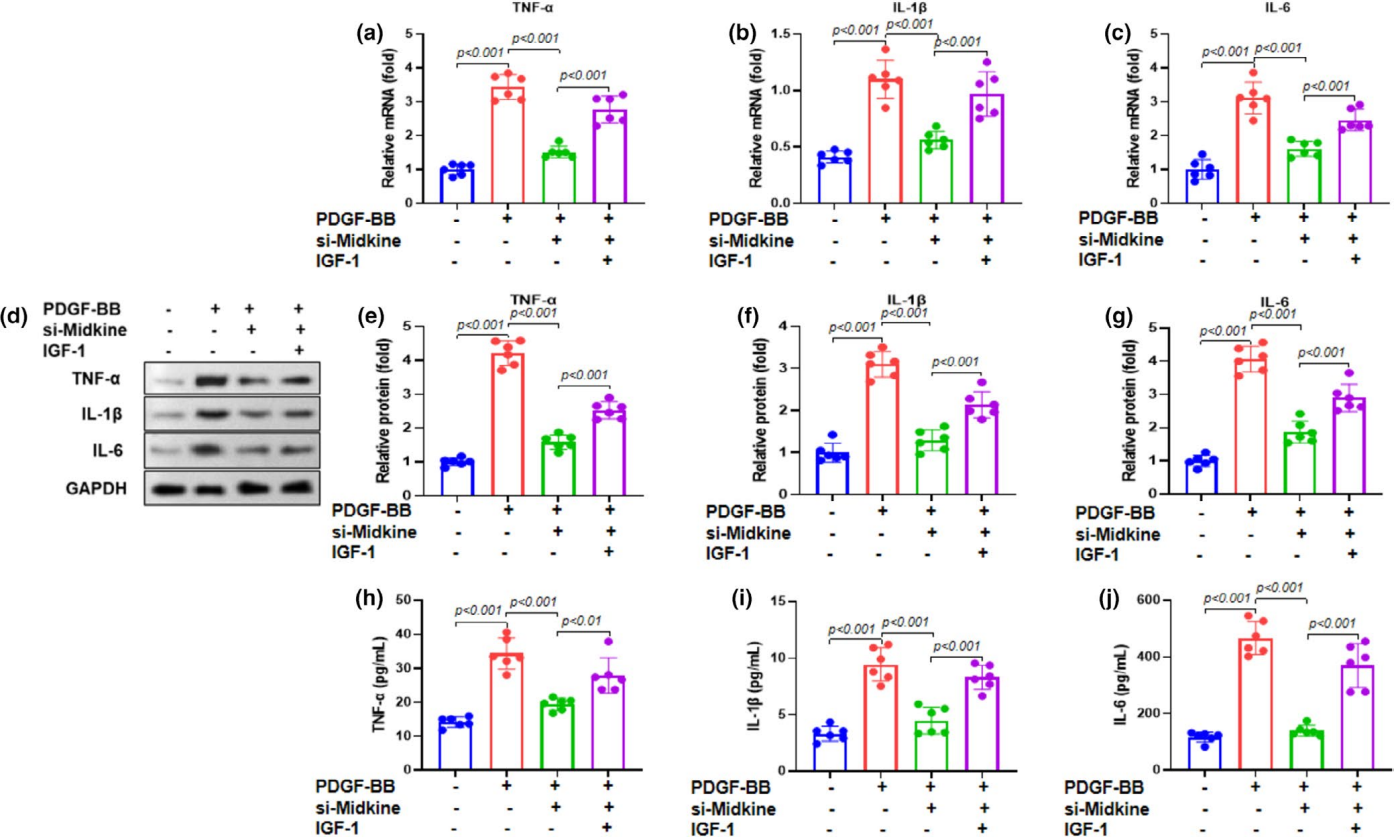
Midkine knockdown suppresses inflammation stimulated by PDGF‐BB. The mRNA expression of pro‐inflammatory cytokines (a) TNF‐α, (b) IL‐1β, and (c) IL‐6 was detected by RT‐qPCR in ASMCs stimulated with 20 ng/mL PDGF‐BB. (d) Protein expression of pro‐inflammatory cytokines was measured using western blotting. Representative bands are shown. Quantification of (e) TNF‐α, (f) IL‐1β, and (g) IL‐6 proteins was performed in ASMCs relative to GAPDH. ELISA was used to detect the levels of (h) TNF‐α, (i) IL‐1β, and (j) IL‐6 in ASMCs supernatants. Data are presented as mean ± SD from at least three independent biological replicates, each performed in six duplicate wells, and analyzed using one‐way ANOVA followed by Bonferroni post‐hoc test.

To explore the role of midkine in PDGF‐BB‐induced inflammation, we assessed the mRNA and protein expression of key pro‐inflammatory cytokines in ASMCs. RT‐qPCR analysis showed that PDGF‐BB (20 ng/mL) markedly increased TNF‐α, IL‐1β, and IL‐6 mRNA levels (Figure 4a–c), whereas midkine knockdown significantly attenuated these increases. Western blot analysis revealed a similar pattern (Figure 4d), with PDGF‐BB treatment elevating TNF‐α, IL‐1β, and IL‐6 protein expression (Figure 4e–g), which was substantially reduced following midkine silencing. Consistent with these results, ELISA demonstrated that PDGF‐BB stimulation enhanced the secretion of TNF‐α, IL‐1β, and IL‐6 into ASMC supernatants (Figure 4h–j), and this increase was markedly suppressed by midkine knockdown. Collectively, these findings indicate that midkine is a key mediator of PDGF‐BB‐induced inflammatory responses in ASMCs, and its silencing effectively inhibits the production and release of proinflammatory cytokines.

### 3.5 Midkine knockdown is associated with reduced glycolysis and altered PI3K/Akt pathway‐related protein expression

**FIGURE 5:**
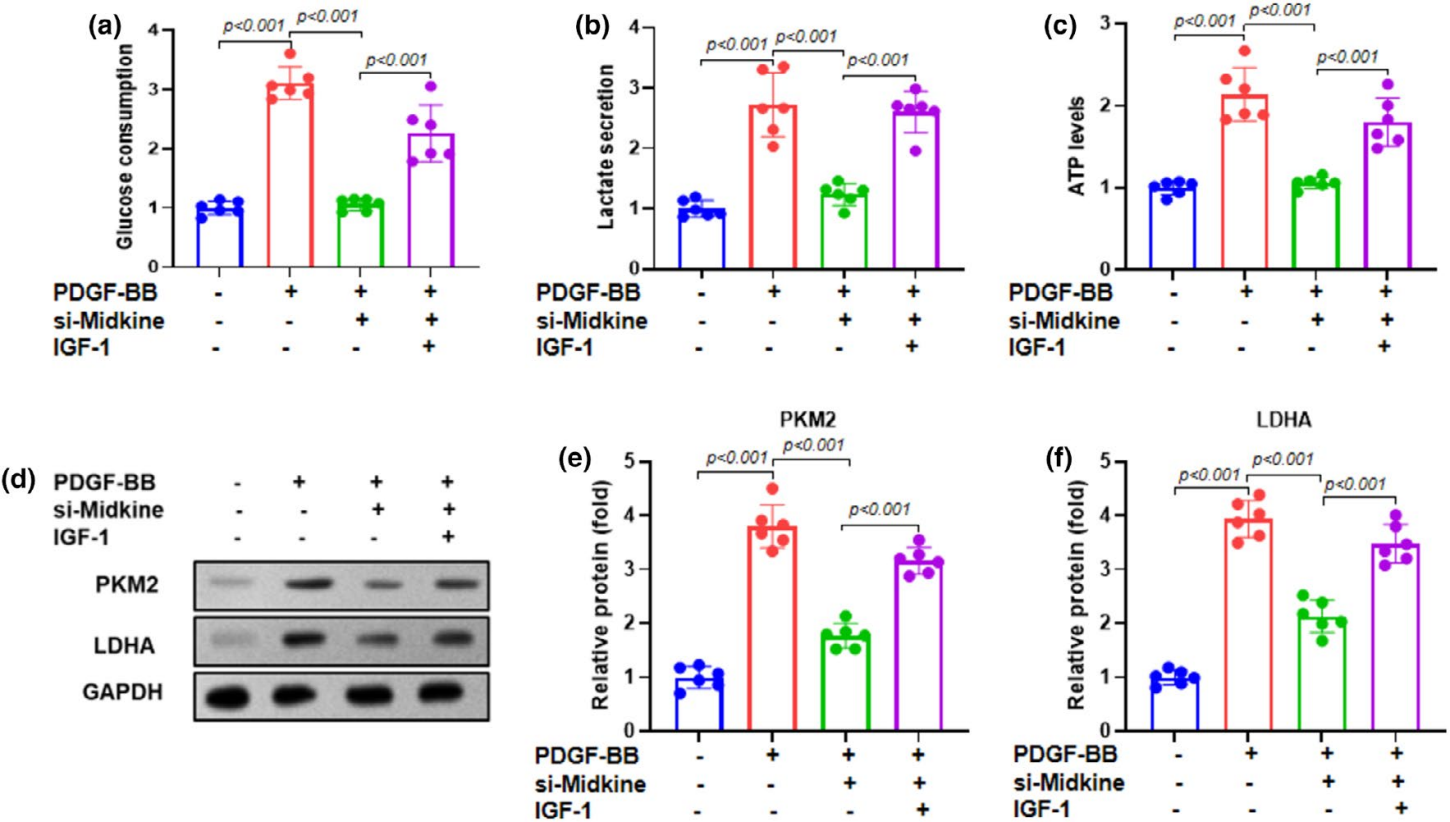
Midkine knockdown suppresses glycolysis via the PI3K/Akt pathway. Glycolysis‐related indicators were measured in the lysate of ASMCs by measuring (a) glucose consumption, (b) lactate secretion, and (c) ATP levels. (d) Representative bands of glycolysis‐related proteins obtained by western blotting. (e, f) Quantification of PKM2 and LDHA levels in ASMCs. Data are presented as mean ± SD from at least three independent biological replicates, each performed in six duplicate wells, and analyzed by one‐way ANOVA followed by Bonferroni post‐hoc test.

To investigate the role of midkine in glycolysis, we assessed key glycolysis‐related indicators in ASMCs following midkine knockdown. Midkine knockdown significantly decreased glucose consumption, lactate secretion, and intracellular ATP levels compared to control cells, indicating a reduction in the glycolytic flux (Figure 5a–c). Western blot analysis revealed that midkine knockdown reduced the expression of key glycolytic enzymes, including pyruvate kinase M2 (PKM2) and lactate dehydrogenase A (LDHA) (Figure 5d). Densitometric quantification confirmed a significant decrease in PKM2 and LDHA protein levels (Figure 5e,f). These results suggest an association between midkine expression and glycolytic activity, potentially involving the PI3K/Akt pathway.

## 4 DISCUSSION

Our findings demonstrate that midkine levels are significantly elevated in the serum of children with asthma and that PDGF‐BB stimulation increases midkine expression in human ASMCs, promoting proliferation. These observations are consistent with the hyperproliferation of ASM in asthma. Indeed, ASMC proliferation has long been established as more pronounced in asthmatics than in healthy controls, both in histological samples and in vitro studies (Johnson et al., 2001). These results extend our current understanding by implicating midkine as a novel contributor to the proliferation axis. Other pathways influencing ASMC proliferation include diacylglycerol kinase‐mediated signaling via mTOR and cyclin D1 activation (Hernandez‐Lara et al., 2022) and signaling through GPCRs, RTKs, and other mitogens mediated through ERK and PI3K cascades (Pelaia et al., 2008; Tu et al., 2025). Midkine‐mediated effects appear to operate primarily through the PI3K/Akt pathway, positioning midkine as an upstream modulator of this critical proliferative signal.

The PI3K/Akt pathway plays a crucial role in cell survival, proliferation, and metabolism (Porta et al., 2014). Our results demonstrated that midkine knockdown significantly suppressed PDGF‐BB‐induced phosphorylation of PI3K and Akt, indicating that midkine modulates this signaling pathway. Similar findings have been reported in cancer cells, where midkine activates the PI3K/Akt pathway to promote cell survival (Muramatsu, 2014). The fact that IGF‐1, a PI3K/Akt activator, partially rescued the effects of midkine silencing further supports the involvement of midkine in this signaling cascade.

Midkine silencing significantly reduced the migratory capacity of ASMCs and downregulated the expression of key extracellular matrix (ECM) proteins, collagen I and fibronectin. These outcomes reinforce the role of midkine in airway remodeling. Increased ASM mass and ECM deposition are hallmarks of airway remodeling in patients with asthma (Hernandez‐Lara et al., 2022). Midkine has also been implicated in another airway disease, COPD, in which the midkine‐Notch2 axis drives ASMC proliferation and remodeling, suggesting shared pathogenic mechanisms between COPD and asthma (Deng et al., 2022). Moreover, midkine silencing attenuated PDGF‐BB–induced expression and secretion of pro‐inflammatory cytokines (TNF‐α, IL‐1β, IL‐6). This finding reveals the dual role of midkine in structural remodeling and inflammation. Inflammatory cytokines, such as TNF‐α and IL‐1β, stimulate ASMC proliferation and contribute to the hyperresponsive airway phenotype in asthma (Khan, 2013).

Midkine knockdown led to a marked reduction in glucose consumption, lactate secretion, and intracellular ATP production in ASMCs, accompanied by the downregulation of key glycolytic enzymes (PKM2 and LDHA). This suggests that midkine contributes to metabolic reprogramming toward enhanced glycolysis in ASMCs. Metabolic rewiring, specifically increased aerobic glycolysis, also known as the Warburg effect, is gaining recognition as a component of asthma pathophysiology. Both immune and structural cells in asthma exhibit elevated glycolytic flux, which supports inflammation and airway remodeling (Goretzki et al., 2023; Luo & Ge, 2025; Lv et al., 2025). For example, glycolysis inhibition ameliorated airway inflammation and hyperreactivity in mouse models of asthma (Yu et al., 2022). Our findings suggest that midkine is a novel regulator of glycolysis in ASMCs, possibly through its modulation of PI3K/Akt signaling, which is known to promote glycolytic enzyme expression and glucose transporter trafficking (Porta et al., 2014).

Several groups have investigated PDGF‐BB signaling in ASMCs and consistently identified PI3K/Akt as the core proliferative/migratory axis. Modulators range from microRNAs (e.g. miR‐18a) to secreted proteins (e.g. CST1 and CIAPIN1), which restrain or potentiate PDGF‐BB responses by modulating PI3K/Akt activity. Our findings are consistent with these studies, but we add that midkine is required to achieve the full amplitude of PDGF‐BB–Akt signaling, positioning midkine as a tractable upstream target. In parallel, studies targeting matrix remodeling enzymes (MMP‐9) or vesicular trafficking (RAB11A) reported suppression of PDGF‐BB–evoked ECM accumulation, mirroring our collagen I/fibronectin results and reinforcing the concept that blocking “feed‐forward” remodeling nodes yields broad benefits (Gong et al., 2022; Liu et al., 2023; Yang et al., 2021; Zhu et al., 2025).

Concerning inflammation, prior work established ASM as a source of IL‐6, RANTES, and other mediators under TNF‐α/IL‐1β or viral‐mimetic stimulation; however, little is known about the contribution of midkine itself to PDGF‐driven cytokine output. Our data fill this gap by showing that midkine knockdown attenuates PDGF‐BB–induced TNF‐α, IL‐1β, and IL‐6 at the mRNA, protein, and secreted levels, consistent with midkine's recognized ability midkine to amplify PI3K/Akt‐NF‐κB–linked inflammatory signaling (Clarke et al., 2009; Damera & Panettieri Jr., 2011). In terms of metabolism, recent reviews and experimental reports in human ASM indicate that heightened glycolysis fuels contractile shortening and remodeling, and that energy stress or glycolytic blockade confers bronchoprotection in ASM. Our observation that midkine knockdown lowers PKM2/LDHA and glycolytic flux dovetails with this concept and suggests a mechanistic link between growth factor signaling and bioenergetics in ASM (Lv et al., 2025; Xu et al., 2023).

The convergence of our proliferation/migration, ECM, cytokine, and metabolic readouts on the midkine–PI3K/Akt axis supports a model in which midkine is a master amplifier of PDGF‐BB signaling in ASMCs. Because airway remodeling (including increased ASM mass and subepithelial matrix) is a key determinant of fixed airflow limitation and disease persistence, targeting midkine could attenuate multiple remodeling outputs simultaneously. Small‐molecule or biologic midkine antagonists have been explored in other diseases, and the present data justify evaluating midkine inhibition or interrupting its downstream PI3K/Akt coupling as a strategy to blunt ASM‐centric remodeling and inflammation in asthma (Hough et al., 2020; Joseph & Tatler, 2022).

## 5 LIMITATIONS

Several limitations of this study should be acknowledged: (1) while the investigation concentrated on the PI3K/Akt pathway, PDGF‐BB and midkine may also activate alternative signaling cascades, such as the MAPK, NF‐κB, or JAK/STAT pathways. (2) The research was conducted using ASMCs in vitro, which may not fully replicate the intricate microenvironment of asthma or chronic obstructive pulmonary disease (COPD) in vivo. (3) The physiological significance of midkine in airway remodeling remains uncertain in the absence of studies involving animal models or human tissues. (4) This study does not directly confirm that midkine regulates glycolysis via the PI3K/Akt pathway, as no inhibitor experiments were performed. Therefore, further studies using PI3K/Akt pathway inhibitors are required to validate this mechanistic relationship.

## 6 CONCLUSION

**FIGURE 6:**
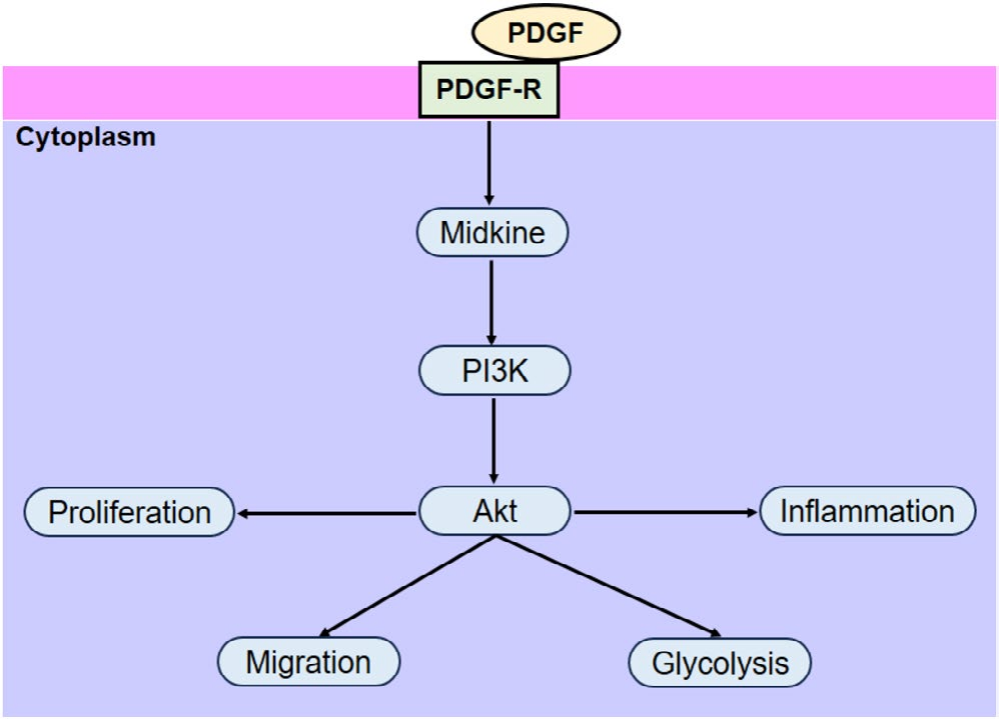
Schematic representation of the role of midkine in the proliferation, migration, inflammation, and glycolysis of ASMCs.

Midkine levels are significantly elevated in asthma and promote PDGF‐BB‐induced ASMC proliferation, migration, ECM production, inflammatory cytokine release, and glycolysis via the PI3K/Akt signaling pathway. Silencing midkine effectively suppresses these pathological processes, including the phosphorylation of PI3K/Akt, expression of collagen I, fibronectin, proinflammatory cytokines, and glycolytic enzymes (PKM2, LDHA) (Figure 6). These findings highlight midkine as a critical mediator of airway remodeling and inflammation, suggesting its potential as a therapeutic target for asthma management.

## AUTHOR CONTRIBUTIONS

**Tianxu Yong**: Conceptualization, Data curation, Investigation, Methodology, Writing‐original draft Preparation. **Jun Shi, Wen Li**: Data curation, Investigation, Methodology, Formal analysis, Writing‐review and editing. **Yanfang Guo**: Writing‐review and editing, Supervision, Project administration, Methodology, Investigation, Funding acquisition, Formal analysis, Data curation.

## FUNDING INFORMATION

This study was supported by Pudong New Area Health Commission's Important Weak Discipline Construction Project (PWZbr2022_06).

## ETHICS STATEMENT

The Ethics Committee of Shanghai Pudong New District Gongli Hospital approved (GLYY1s2024‐052) this study. The authors followed all standard protocols in accordance with the 1964 Declaration of Helsinki. All participants' parents or legally authorized guardians provided written informed consent to participate in the study.

## COMPETING OF INTEREST STATEMENT

The authors report no conflicts of interest in this work.

## Supporting information


Data S1.

Data S2.

## Data Availability

Due to confidentiality issues, the datasets generated and/or analyzed during the current work are not publicly available but are available from the corresponding author upon reasonable request.
