# Supplementary material for: Midkine promotes PDGF‐BB‐induced proliferation, migration, and glycolysis of airway smooth muscle cells via the PI3K Akt pathway
Source: Physiol Rep. 2025 Sep 19;13(18):e70553. doi: 10.14814/phy2.70553 (PMC12447002; doi:10.14814/phy2.70553)
Supplement: Supplementary file 1 — Data S1. [file PHY2-13-e70553-s001.pdf]

Midkine promotes PDGF-BB-induced proliferation, migration, and glycolysis of airway smooth muscle cells through the PI3K Akt pathway

Tianxu Yong[1], Jun Shi[1], Wen Li[2], Yanfang Guo[1]✉

Supplementary Fig. S1

Original uncropped raw images of western blot panel used in **Figure 1C**

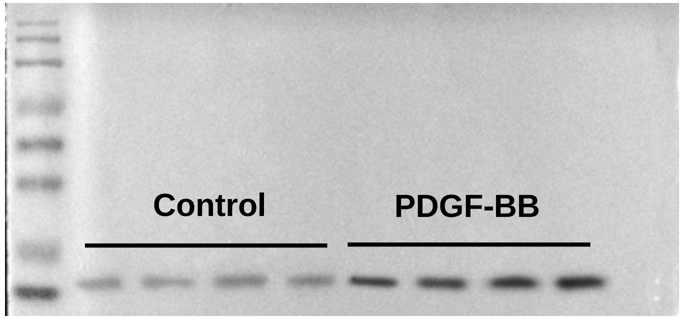

13.3 kDa

Midkine

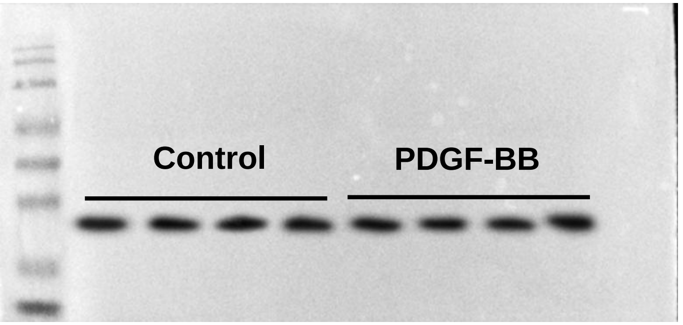

36 kDa

GAPDH

Supplementary Fig. S2

Original uncropped raw images of western blot panel used in **Figure 1G**

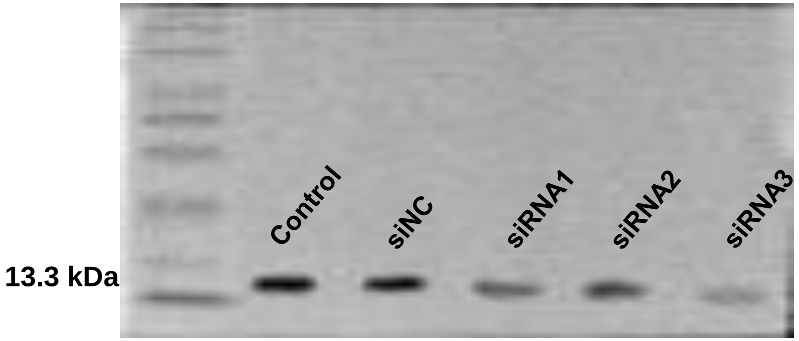

Midkine

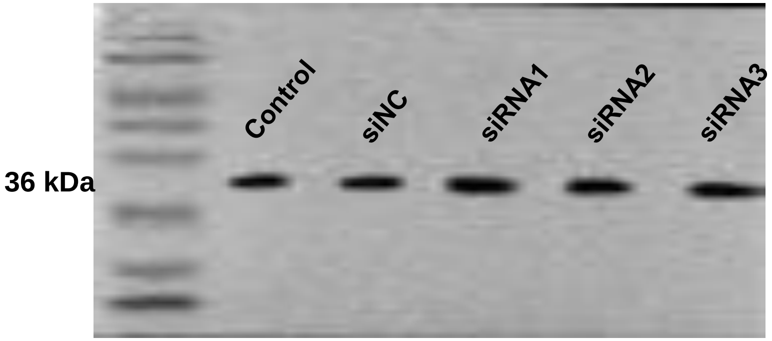

GAPDH

Supplementary Fig. S3

Original uncropped raw images of western blot panel used in **Figure 2A**

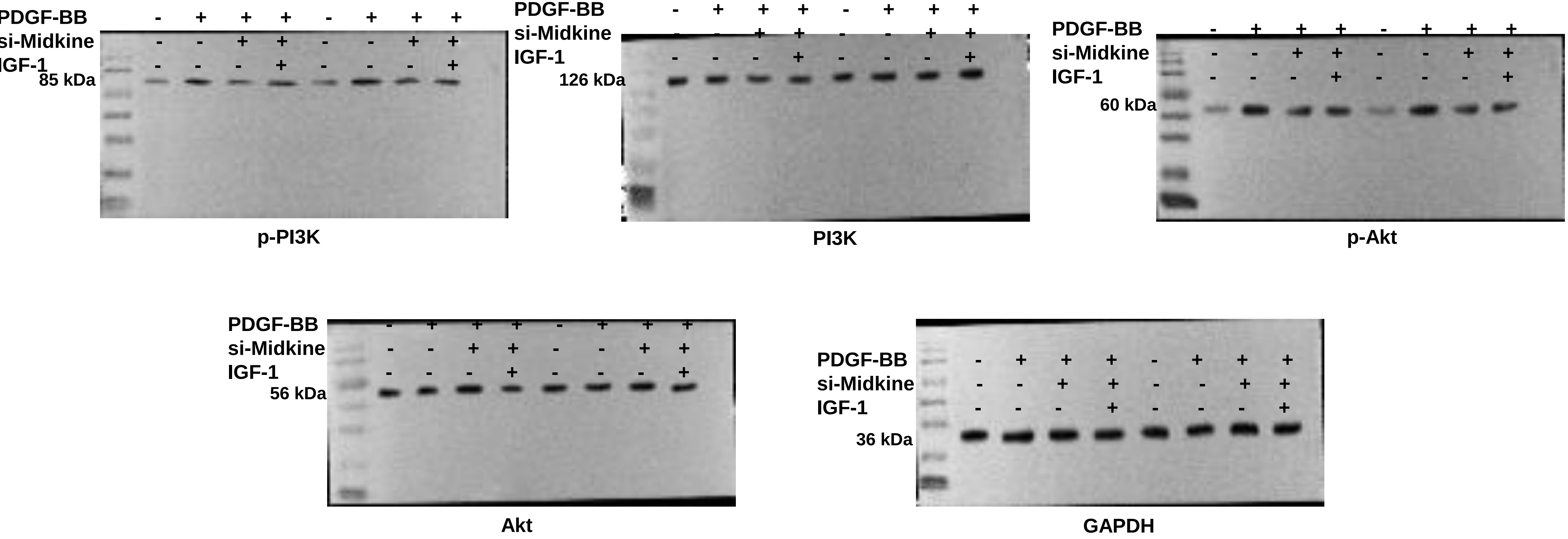

Supplementary Fig. S4

Original uncropped raw images of western blot panel used in **Figure 3E**

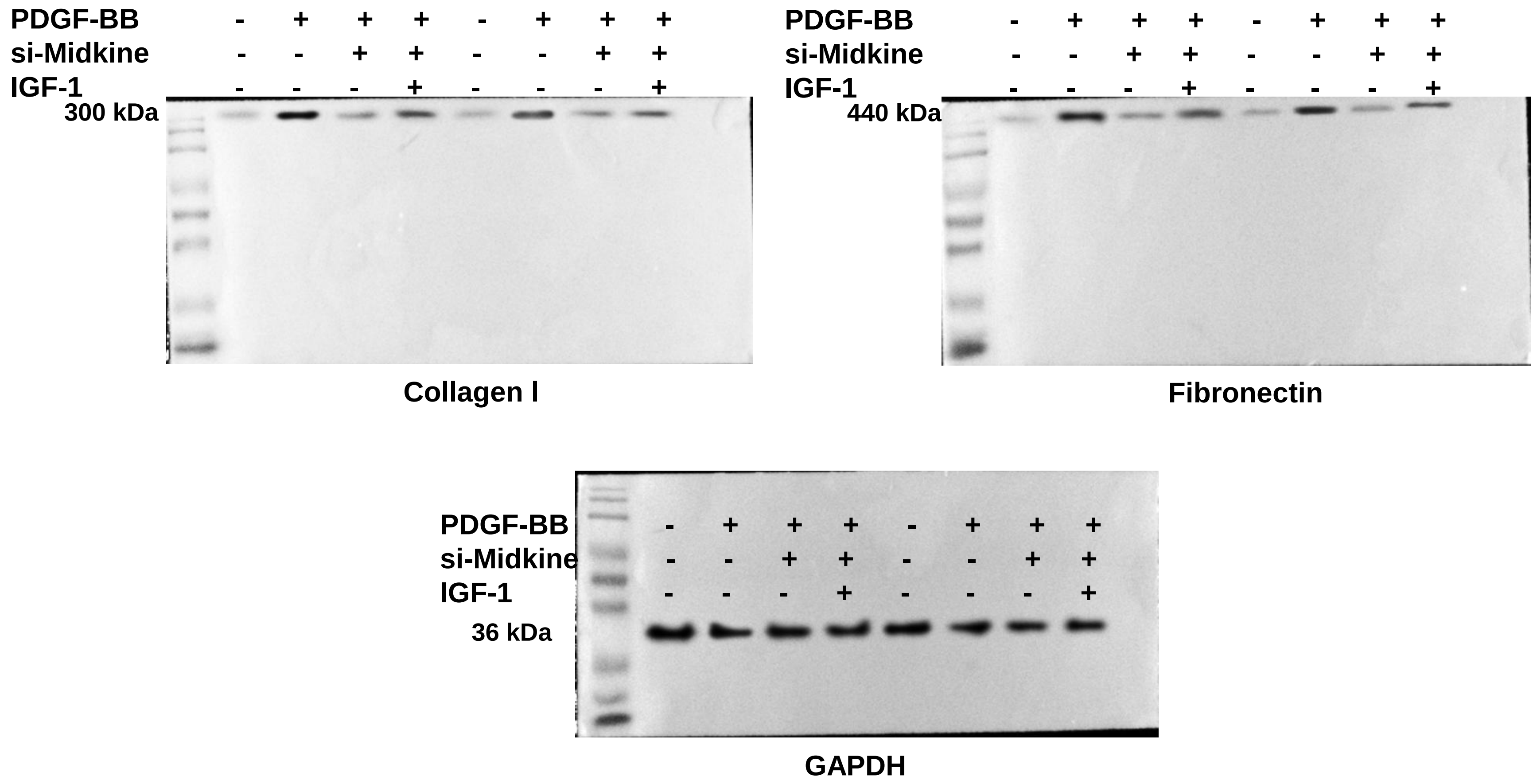

Supplementary Fig. S5

Original uncropped raw images of western blot panel used in **Figure 4D**

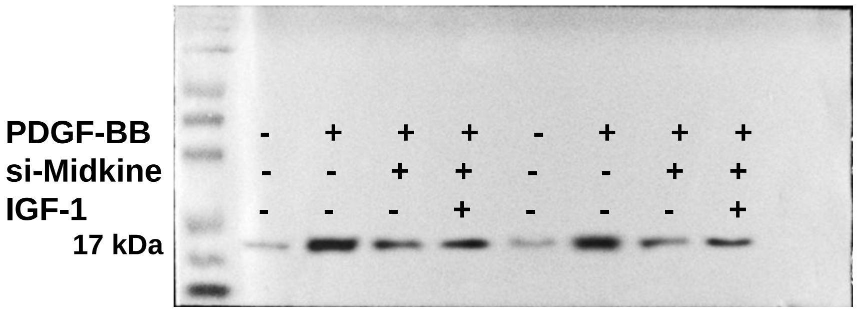

TNF-α

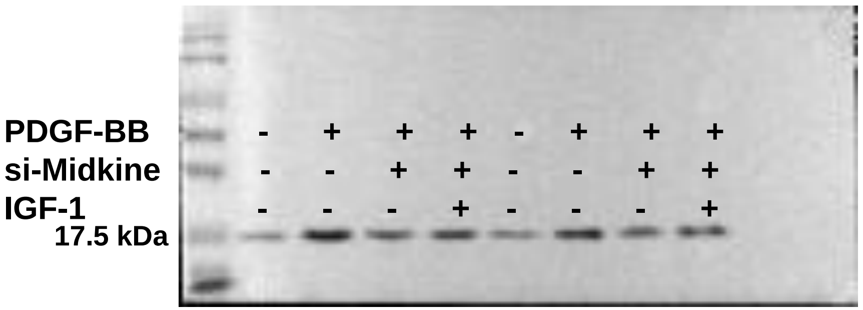

IL-1β

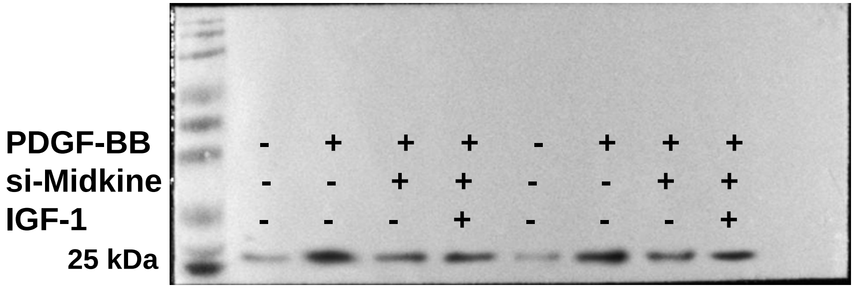

IL-6

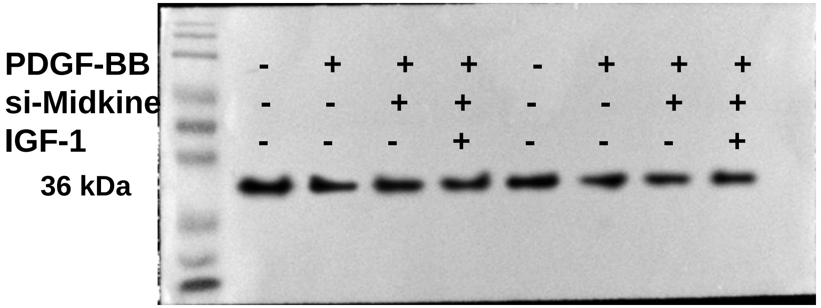

GAPDH

Supplementary Fig. S6

Original uncropped raw images of western blot panel used in **Figure 5D**

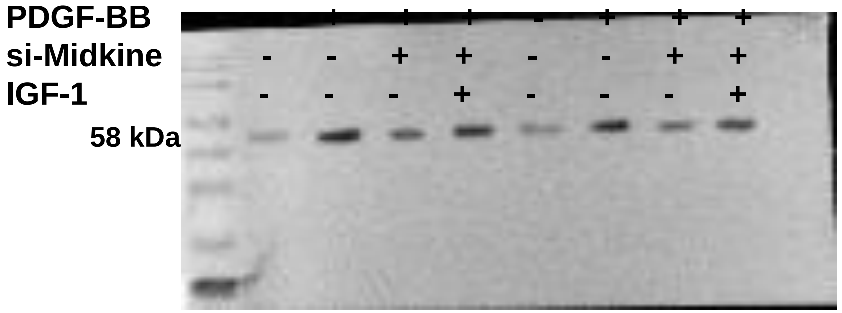

PKM2

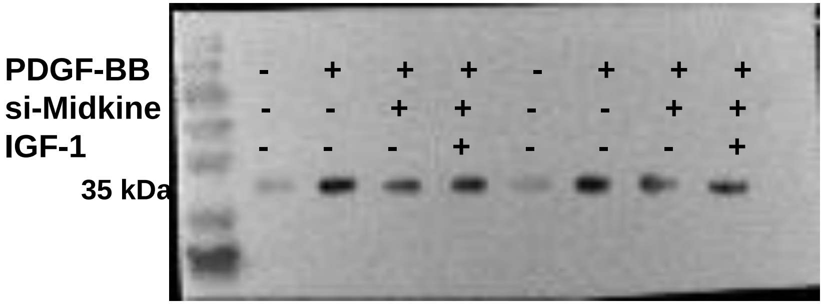

LDHA

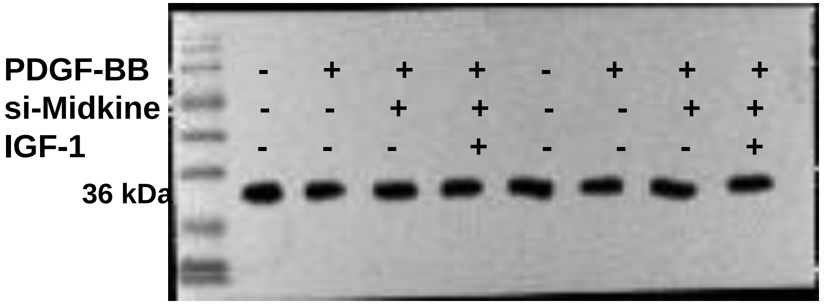

GAPDH
