# Supplementary material for: Midkine promotes PDGF‐BB‐induced proliferation, migration, and glycolysis of airway smooth muscle cells via the PI3K Akt pathway
Source: Physiol Rep. 2025 Sep 19;13(18):e70553. doi: 10.14814/phy2.70553 (PMC12447002; doi:10.14814/phy2.70553)
Supplement: Supplementary file 2 — Data S2. [file PHY2-13-e70553-s002.pdf]

Table S1. Characteristics of the healthy controls and asthma patients

Clinical parameters	Control group (n = 20)	Asthma group (n = 20)	P-value
Age (years)	6.7±2.4	7.1±1.9	0.609
Sex (male, %)	12 (60%)	11 (55%)	0.756
BMI (kg/m^2)	18.85±1.80	19.38±1.86	0.367
FEV1 (L)	2.22±0.4	1.62±0.32	<0.001
FEV1/FVC (%)	83.93±2.85	73.84±3.79	<0.001
PEF (L/min)	85.76±2.73	65.81±5.79	<0.001
Eosinophil count (per/μL)	149.77±22.54	457.95±100.41	<0.001
Total IgE (IU/mL)	143.67±16.1	240.81±68.95	<0.001
FeNO (ppb)	21.83±2.62	37.61±5.60	<0.001
TNF-α (pg/mL)	22.28±3.60	32.58±7.71	<0.001
IL-1β (pg/mL)	61.75±8.55	112.67±20.17	<0.001
IL-4 (pg/mL)	44.54±4.97	78.99±10.72	<0.001
TGF-β1 (pg/mL)	26.96±4.43	44.73±6.65	<0.001
MMP-9 (ng/mL)	84.53±13.98	124.68±17.63	<0.001
SDF-1α (pg/mL)	379.62±86.09	568.62±81.67	<0.001
Midkine (pg/mL)	764.99±127.44	1558.47±302.04	<0.001

Abbreviation: BMI, body mass index; FEV1, forced expiratory volume in one second; FVC, forced vital capacity; PEF, peak expiratory flow rate; IgE, immune globulin E; FeNO, exhaled nitric oxide; TNF-α; tumor necrosis factor-α; IL-1β, interleukin 1β; IL-4, interleukin 4; TGF-β1, transforming growth factor-β1; MMP-9, matrix metalloproteinases-9; SDF-1α, stromal-derived factor 1α.
